# Enhanced Abandoned Object Detection through Adaptive Dual-Background Modeling and SAO-YOLO Integration

**DOI:** 10.3390/s24206572

**Published:** 2024-10-12

**Authors:** Lei Zhou, Jingke Xu

**Affiliations:** 1 College of Computer Science and Engineering, Shenyang Jianzhu University, Shenyang 110168, China; zhoulei@stu.sjzu.edu.cn; 2 Liaoning Province Big Data Management and Analysis Laboratory of Urban Construction, Shenyang 110168, China; 3 Shenyang Branch of National Special Computer Engineering Technology Research Center, Shenyang 110168, China

**Keywords:** abandoned object detection, dual-background model, YOLO, local feature extraction

## Abstract

Abandoned object detection is a critical task in the field of public safety. However, existing methods perform poorly when detecting small and occluded objects, leading to high false detection and missed detection rates. To address this issue, this paper proposes an abandoned object detection method that integrates an adaptive dual-background model with SAO-YOLO (Small Abandoned Object YOLO). The goal is to reduce false and missed detection rates for small and occluded objects, thereby improving overall detection accuracy. First, the paper introduces an adaptive dual-background model that adjusts according to scene changes, reducing noise interference in the background model. When combined with an improved PFSM (Pixel-based Finite State Machine) model, this enhances detection accuracy and robustness. Next, a network model called SAO-YOLO is designed. Key improvements within this model include the SAO-FPN (Small Abandoned Object FPN) feature extraction network, which fully extracts features of small objects, and a lightweight decoupled head, SODHead (Small Object Detection Head), which precisely extracts local features and enhances detection accuracy through multi-scale feature fusion. Finally, experimental results show that SAO-YOLO increases mAP@0.5 and mAP@0.5:0.95 by 9.0% and 5.1%, respectively, over the baseline model. It outperforms other advanced detection models. Ultimately, after a series of experiments on the ABODA, PETS2006, and AVSS2007 datasets, the proposed method achieved an average detection precious of 91.1%, surpassing other advanced methods. It significantly outperforms other advanced detection methods. This approach notably reduces false and missed detections, especially for small and occluded objects.

## 1. Introduction

As a critical task in the field of public safety, abandoned object detection primarily involves two key steps: (1) identifying the location of suspicious objects, and (2) classifying the identified suspicious objects to further confirm whether they are abandoned objects. Before the significant advancements in deep learning technology, abandoned object detection mainly relied on traditional computer vision methods to construct background models for filtering abandoned objects. These methods include frame difference, Gaussian Mixture Model (GMM) [1,2], and ViBe algorithm [3,4]. These techniques use background subtraction to distinguish between the background, moving objects, and stationary objects [5,6], thereby filtering out stationary foreground objects. Among them, the Gaussian Mixture Model (GMM) is a statistical method based on the distribution of pixel values, modeling each pixel value through the weighted processing of multiple Gaussian distributions. It is known for its low computational cost and strong noise resistance.

**Figure 1:**
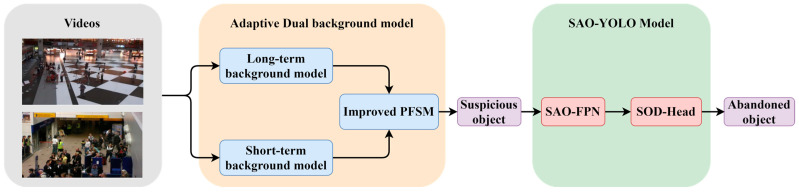
Flowchart of the abandoned object detection method.

In recent years, deep learning-based object detection methods have made significant progress, with convolutional neural networks (CNNs) playing a crucial role in object detection due to their high detection accuracy. Convolutional neural network-based object detection models are primarily divided into two categories. The first category is single-stage models, which use CNNs to directly predict the category and location of objects. These models perform classification and regression in a single pass, omitting the candidate box generation stage, resulting in faster detection speeds. Representative models include the YOLO series [7,8,9,10,11,12] and SSD [13]. The second category is two-stage models, such as Fast R-CNN [14], Faster R-CNN [15], and Mask R-CNN [16]. These models first use CNN to generate candidate regions and then perform classification and regression on these regions, leading to slower detection speeds but higher accuracy. These techniques are widely applied in various fields, including video surveillance, security, industry, and agriculture. As a result, the performance of abandoned object detection methods has significantly improved under two mainstream approaches: (1) abandoned object detection methods based on deep learning technology, offering excellent detection accuracy, and (2) methods that combine traditional computer vision techniques with deep learning technology, balancing detection speed and accuracy. Abandoned object detection methods that integrate computer vision and deep learning technologies can maintain high detection accuracy while achieving faster detection speeds, making them highly valuable for practical applications and the current mainstream approach to abandoned object detection [17,18,19,20,21]. However, in existing public space video surveillance, accurate detection of discarded objects remains a challenge due to the frequent presence of small objects that are obscured, as shown in Figure 1. These objects typically demonstrate characteristics such as few pixels, lacking feature information, occlusion, and truncation, which result in significant false positives and false negatives.

Initially, the videos are input into the long-term and short-term dual-background model to establish the background model. Then, the improved PFSM model is used to accurately extract suspicious objects (static foreground objects), which are subsequently fed into the SAO-YOLO model for further classification, ultimately outputting the detected abandoned objects. The small objects (up) in the figure are video frames from the ABODA dataset, while the occluded objects (down) are video frames from the PETS2006 dataset. In previous approaches to abandoned object detection, Lin et al. [22] proposed a long-term and short-term dual-background model for constructing the background model and introduced a Pixel-based Finite State Machine (PFSM) model to extract suspicious objects. Shayam et al. [19] further enhanced this by employing the sViBe method to construct long-term and short-term background models, combined with deep learning techniques like SSD to detect humans and suspicious object types. Ilya et al. [23] introduced Network Output Background Subtraction (NOBS), aiming to obscure the classification of detected abandoned objects by training on objects of unspecified categories, thus increasing the method’s applicability. Lin et al. [17] proposed the YOLO-G network model, which enhances feature extraction capabilities to improve the overall detection accuracy of abandoned object detection methods. Saluky et al. [21] integrated the dual-background model with the advanced YOLO-NAS model to enhance the precision and robustness of abandoned object detection methods. Although these methods have improved overall detection accuracy by refining foreground-background models and enhancing deep learning network models, they do not specifically objects improvements for any particular type of abandoned object. As a result, their performance in detecting small and occluded objects in abandoned object detection scenarios is suboptimal.

In recent years, many scholars have conducted research on small objects. Due to the characteristics of small objects and occluded objects, such as having few pixels and unclear texture features, the detection performance of the general object detection models mentioned earlier is inadequate, making them less effective in complex abandoned object scenarios. To better extract the feature information of small objects, Li et al. [24] proposed combining a Decoupled Head (DH) with a Mixed Attention Module (MAM), integrating the MAM module into the Head to allow each branch to learn the feature information needed for classification and regression. Xiong et al. [25] embedded a subspace attention mechanism into the model, suppressing background interference by integrating multi-scale feature information. Zeng et al. [26] introduced a Spatial Coordination Attention (SCA) module to enhance the model’s ability to locate small objects and adopted an SEB module to reduce noise interference. Gao et al. [27] argued that under limited resource conditions, the model should be optimized to maximize accuracy while efficiently using resources by embedding C3 CrossConv into the model to reduce overall parameters and computation while adding a small object layer and Global Attention Mechanism (GAM) [28] to ensure overall detection accuracy. Zhou et al. [29] proposed a feature fusion and search algorithm based on CBAM [30] and an efficient network, enabling diversified feature extraction and enhancing the model’s ability to detect small objects. Tong et al. [31] introduced a Single-layer Depth Feature Learning Module (DFLM) to activate a broader range of feature information and incorporated a Feature Fusion Block (FFB) to merge shallow feature information, improving the extraction capability for small object features. Yang et al. [32] proposed a Feature Enhancement Module (FEM) and a Deep Feature Fusion Module (DPPM) to increase accuracy by expanding the receptive field of the feature prediction layer and retaining more detailed information. Huynh et al. [33] introduced a new SR module that extends the input into three feature maps, synthesizing a super-resolution feature map to compensate for the lack of characteristic information in small objects, which effectively improves detection performance but also significantly increases computational scale and reduces detection speed. These methods primarily enhance detection accuracy by improving the network’s feature extraction capabilities for small objects and enhancing the fusion of small object feature information.

Inspired by the aforementioned research, during the exploration of the characteristics of small objects and occluded objects, this paper puts forward a set of effective and innovative approaches to enhance the measures for improving the detection accuracy and robustness. The specific testing process is depicted in Figure 1. The following is an analysis of the improvement indicators in the process:

(1) Adaptive double-background model: Through the introduction of impact factors and the establishment of an adaptive learning rate, the sensitivity of the model to noise can be dynamically adjusted, thereby effectively reducing the influence of noise interference. This improvement not only enhances the accuracy of the background model but also strengthens its robustness in complex environments. (2) SAO-FPN feature extraction network: This network enhances the detection performance of the baseline model on small target images by means of multi-layer feature fusion. By pruning redundant modules, it mitigates the information loss during the forward propagation process and enhances the feature representation capability of the model. The improvement of detection accuracy is specifically reflected in a 4.9-percentage-point increase in mAP@0.5 and a 3.0-percentage-point increase in mAP@0.5:0.95, demonstrating the effectiveness of the network in fine-grained feature extraction. (3) SAO-Head decoupling head: To address the issue of insignificant local features of small and occluded objects, the SAO-Head decoupling head is introduced, allowing for the accurate extraction of local features. This improvement of its detection accuracy is reflected in a 0.8-percentage-point increase in mAP@0.5 and a 0.5-percentage-point increase in mAP@0.5:0.95. (4) Recall rate and accuracy rate: Through the application of the new model, the recall rate (R) is raised to 100%, and the accuracy rate (P) is increased to 91.1%. A series of experiments were conducted on the ABODA dataset, PETS2006 dataset, AVSS2007 dataset, and VisDrone dataset.

The abandoned target detection method, which combines the adaptive double-background model and SAO-YOLO, mainly accounts for the noise effect brought about by scene changes through the design of the adaptive double-background model, and is capable of dynamically adjusting the learning rate of the background model, enabling it to adapt to diverse environmental conditions. This ability allows the model to extract real target features more stably in the face of a complex background, thereby reducing false detections and missed detections. The improved PFSM model is based on the enhanced Foreground segmentation model (PFSM), which can identify and extract suspicious static foreground objects more precisely, further enhancing the detection capability of small targets and occluded targets. By utilizing the established adaptive dual-background model, the PFSM model can effectively separate the foreground and background, enhancing the ability to extract target features. In terms of the optimization of the SAO-YOLO model, it can extract the features of small targets and occluded objects more fully by employing a specific feature extraction network such as SAO-FPN. This network design emphasizes the fusion of multi-scale features, that is, to ensure that the key features of small objects can be effectively recognized even at low-resolution inputs. Simultaneously, the design of the SAO-Head decoupling head enables the model to accurately extract features of small objects and partially occluded objects. By processing the features independently, the decoupled head avoids the mutual interference of information, thus improving the accuracy of target recognition.

As can be seen from Figure 1, the main innovation process of this paper can be summarized as follows:

- An adaptive dual-background model is proposed, which adjusts the update rate of the background model based on changes in lighting and target complexity, reducing the interference of external noise in extracting static foreground objects. Additionally, the PFSM model is improved by introducing an occlusion state to enhance the accuracy of detecting occluded objects.
- A multi-scale information fusion network model, SAO-YOLO, is put forward for the detection of small and occluded objects. This model further categorizes the output suspected objects (static foreground objects). The SAO-FPN feature extraction network and the SODHead decoupling head are incorporated into the network model, strengthening the overall feature fusion ability of the model and enhancing the detection accuracy.
- The SAO-FPN feature extraction network is designed to adjust the feature extraction network for small object features. A multi-scale object detection layer is designed for small object images, improving the detection performance of the baseline model on small object images. By pruning redundant modules, the model’s information loss during forward propagation is reduced, and the feature representation capability of the model is enhanced.
- The SODHead decoupling head is designed to fuse the local features extracted from the lower detection layers with the higher-layer features using an embedded LFEM module, improving the accuracy of detecting occluded and small objects at the higher detection layers. The designed LFEM module extracts key local features from the output of the lower feature layer through operations such as cropping, padding, and rearranging, and integrates them with the higher-layer features using a self-attention mechanism to achieve precise feature extraction.
- A series of experiments were conducted on the ABODA dataset, PETS2006 dataset, AVSS2007 dataset, and VisDrone dataset, and the results were analyzed. The results show that the proposed network model significantly improves the detection performance for abandoned objects.

## 2. Proposed Method

### 2.1. Adaptive Dual-Background Model

The dual-background model is an extended version of the background-foreground model, designed for foreground detection in dynamic scene analysis. It primarily consists of two parts: a long-term background model and a short-term background model. The long-term background model operates with a lower learning rate, reducing the update speed of the background, while the short-term background model uses a higher learning rate to accelerate the background updates. The difference in update rates between the two models enables the detection of suspicious objects (static foreground objects).

Based on the dual-background model, this paper presents an adaptive dual-background model. Through the introduction of an adaptive learning rate, the model is capable of automatically adjusting the dual-background model and, in conjunction with an improved PFSM model, attaining precise extraction of suspicious objects.

#### 2.1.1. Establishment of the Adaptive Dual-Background Model

Traditional computer vision methods have small computational scales and fast processing speeds, but they are susceptible to noise interference caused by scene changes. Since small and occluded objects have relatively small sizes, the impact of noise on them is particularly severe. This paper establishes the background model using the proposed Adaptive Gaussian Mixture Model, adjusting the update rate of the background model based on scene changes, which significantly enhances the model’s resistance to interference. The Gaussian Mixture Model represents each pixel by defining K Gaussian distributions to describe its pixel value. The random probability distribution of the current pixel value $X_{t}$ at time t is described as:

(1) $$P(X_{t})=\sum_{j=1}^{K}\omega_{j,t}\eta (X_{t},\mu_{j,t},\sum_{j,t}),$$

(2) $$\eta (X_{t},\mu_{j,t},\sum_{j,t})=\frac{1}{\sqrt{{\left(2\pi \right)}^{n}\left|\sum_{j,t}\right|}}e^{-\frac{1}{2}{\left(X_{t}-\mu_{j,t}\right)}^{T}\sum_{j,t}^{-1}(X_{t}-\mu_{j,t})},$$

In the equation, $P(X_{t})$ represents a Gaussian model; K is the total number of pixels; t indicates the specific time point; j refers to a pixel; $\omega_{j,t}$ is the weight of the j-th Gaussian distribution at time t; $\eta (X_{t},\mu_{j,t},\sum_{j,t})$ is the density of the j-th Gaussian distribution at that moment; $X_{t}$ is the pixel value at time t; $\mu_{j,t}$ is the expected value (mean) of the j-th Gaussian distribution at time t; $\sum_{j,t}$ is the covariance matrix of the j-th Gaussian distribution at that time; n represents the dimensionality of the pixel; and e is the base of the natural logarithms used in the calculations.

The Adaptive Gaussian Mixture Model is divided into two phases: the initial phase and the stable phase. During the initial phase, when the model has just been established, a higher learning rate is required to quickly update the background model and eliminate environmental noise interference. In the stable phase, to reduce noise interference and enhance the model’s adaptability to changes in lighting and object complexity, this paper introduces factors for changes in lighting and object complexity. These factors adjust the influence factor γ, thereby achieving adaptive adjustment of the model’s learning rate, enhancing the accuracy and robustness of the background model detection. The setting of the adaptive learning rate is outlined as follows:

(3) $$\alpha =\left\{\begin{array}{cc} \lambda_{0} & T<T_{0}\\ \lambda_{0}(1-\gamma ) & T\geq T_{0} \end{array}\right.,$$

(4) $$\varepsilon =\frac{N_{obj}}{N_{all}},\delta =1-\frac{H_{t-1}}{H_{t}},$$

(5) $$\gamma =\left\{\begin{array}{cc} \varepsilon \cdot (1+\delta ) & \varepsilon <50\%\\ 0.5\cdot (1+\delta ) & \varepsilon \geq 50\% \end{array}\right.,$$

In this setup, α represents the learning rate; $\lambda_{0}$ is a constant; γ is the influence factor; T denotes the current frame number; $T_{0}$ is the threshold for frame numbers; ε is the proportion of pixels detected as objects in the current frame relative to the total number of pixels in that frame; δ is the lighting change factor; $N_{obj}$ is the number of pixels identified as detection targets in the current frame; $N_{all}$ is the total number of pixels in the current frame; $H_{t-1}$ is the entropy of the previous frame’s image; and $H_{t}$ is the entropy of the current frame’s image.

Specifically, in the long-term background model, the constant $\lambda_{0}$ is usually $\lambda_{0}\in [0.03,0.06]$, and in the short-term background model, it is usually $\lambda_{0}\in [0.1,0.3]$. The proportion of pixels detected as targets in the current frame, denoted by ε, is used to describe the complexity of objects in the current frame. A higher proportion of target pixels indicates greater complexity, which necessitates a lower learning rate. The lighting change factor, δ, is used to assess the change in lighting between the current frame and the previous frame. If there is a significant change in lighting in the current frame, δ is set to 1, prompting an increase in the learning rate to stabilize the background state as much as possible. Once the state stabilizes, δ is set to 0.

#### 2.1.2. Improved PFSM Model

This paper makes improvements based on the PFSM (Pixel-based Finite State Machine) model by adding an occlusion state to further enhance the accuracy of suspicious objects (static foreground objects) detection. Initially, the established long-term and short-term background models are binarized. The binarized image of the long-term background model is labeled as $F_{L}$, and that of the short-term background model is labeled as $F_{S}$. Based on the different binary information, the state transitions are made, and the binary representation of pixel i at time t is determined as follows:

(6) $$S_{i,t}=F_{L}(i)(t)F_{S}(i)(t),$$

In the equation, $F_{L}(i)(t),F_{S}(i)(t)\in \{0,1\}$, $S_{i,t}$ represents the current state of pixel $F_{L}(i)(t)$ within the long-term background model, and $F_{S}(i)(t)$ within the short-term background model. When $F_{L}(i)(t)$ is 1, it indicates that the current pixel is recognized as a foreground pixel under the long-term background model. Conversely, when $F_{L}(i)(t)$ is 0, it indicates that the pixel is part of the background in the long-term model. Similarly, when $F_{S}(i)(t)$ is 1, the pixel is recognized as a foreground pixel under the short-term background model, and when $F_{S}(i)(t)$ is 0, it is part of the background in the short-term model. Therefore, the value of $S_{i,t}$ can be one of four possible states, which are as follows:

1. $S_{i,t}=00$ indicates that at time t, the pixel is a static background pixel in both the long-term and short-term background models.
2. $S_{i,t}=01$ indicates that at time t, the pixel is in a state of being occluded by other objects.
3. $S_{i,t}=10$ indicates that at time t, the pixel is a static foreground pixel.
4. $S_{i,t}=11$ indicates that at time t, the pixel is a dynamic foreground pixel.

**Figure 2:**
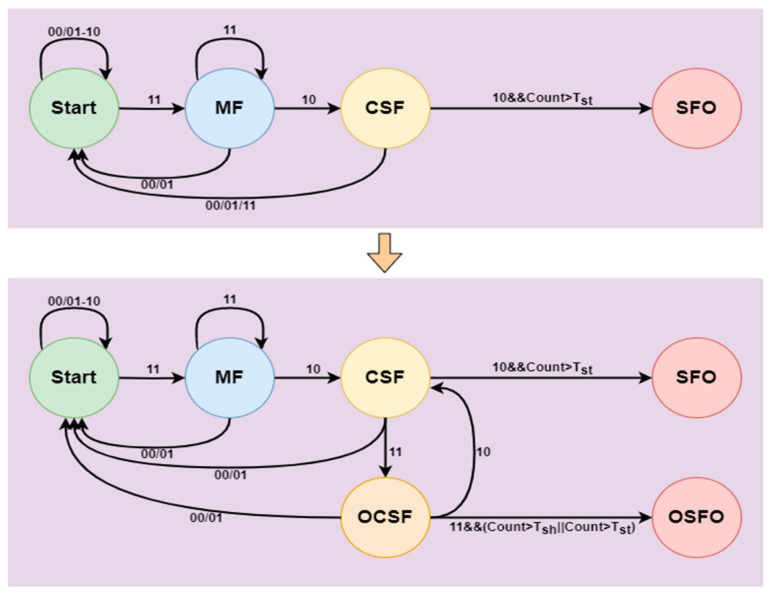
The improved PFSM model.

If only Equation (5) is used for detecting static foreground objects, the judgment relies solely on the pixel state at the current moment, without a reasonable connection to the context. Therefore, its detection results are inaccurate. This paper improves the Finite State Machine (FSM) model by transitioning states based on different state information and introducing an occlusion state. The improved pixel-based Finite State Machine model (PFSM) is proposed to accurately detect static foreground objects, as shown in Figure 2. The newly introduced occlusion state refers to the state where the detected object, after being converted into a candidate static foreground object, is subsequently occluded by another object.

The state transition information of pixels at different time points is used to construct a sequence pattern for identifying the current state of each pixel. Through this pattern, suspicious objects (static foreground objects) are detected, where MF represents Moving Foreground, CSF represents Candidate Static Foreground, OCSF represents Occluded Candidate Static Foreground, SFO represents Static Foreground Object, and OSFO represents Occluded Static Foreground Object. The state information from the background model is input into the improved PFSM model, which outputs the suspicious objects.

In the improved PFSM model, two timers, Count1 and Count2, as well as time thresholds $T_{st}$ and $T_{sh}$, are utilized. Count1 represents the time a foreground object remains stationary, and Count2 represents the duration for which a candidate static foreground object is occluded. The threshold $T_{st}$ is the time required for a static foreground object to be recognized as an abandoned object, while $T_{sh}$ represents the time needed for the object occluding the abandoned item to be considered part of the background by the short-term background model.

The process commences with data acquisition, employing cameras or sensors to capture consecutive frames of images in real time and store each frame for subsequent processing. Subsequently, the acquired images undergo preprocessing, encompassing de-noising, normalization, and gray-scale conversion, to eliminate noise and standardize the image format. Then, multiple states (such as moving foreground MF, candidate static foreground CSF, etc.) are stipulated for each pixel, and these states are initialized in the first frame based on the background modeling algorithm. The state transition matrix is constructed to document the variations of pixel state at different time points, and the sequence pattern is extracted to determine the current state of each pixel. Next, the state information is input into the enhanced PFSM model for analysis. Based on the input information, the model updates the state of each pixel and distinguishes the static foreground object and the occluded static foreground object. The output suspicious objects are labeled in the image and stored for subsequent analysis. To enhance the accuracy of the detection results, post-processing of the suspicious objects is conducted, including morphological operations and region merging. Finally, the test results are presented via visual tools, and the model parameters or state transition rules are adjusted in accordance with the evaluation feedback to optimize the subsequent test performance and guarantee the effectiveness and reliability of the entire process.

### 2.2. Deep Learning Network Model SAO-YOLO

**Figure 3:**
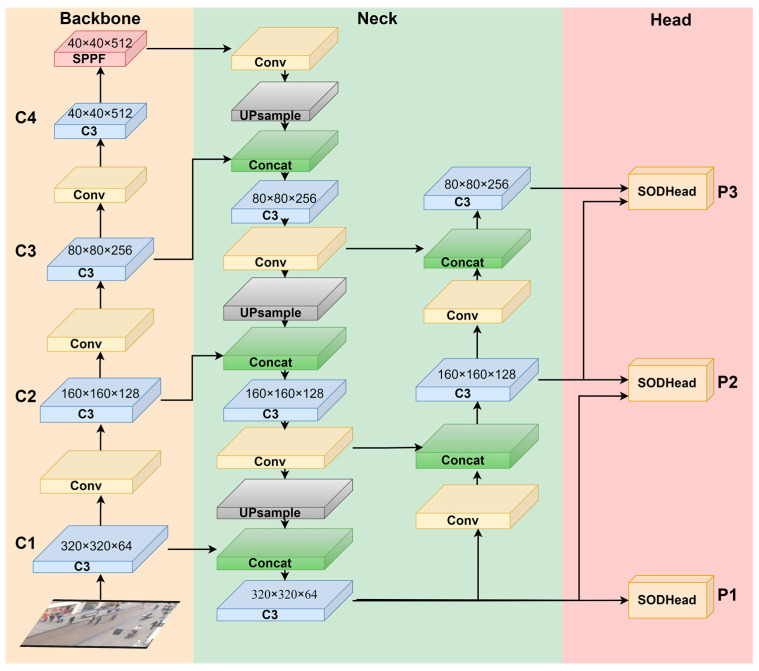
SAO-YOLO structure diagram. (The model primarily consists of three parts: backbone, neck, and head. Improvements in the backbone and neck are achieved through the proposed SAO-FPN structure, which enhances detection accuracy by adjusting model depth and increasing the scale of branches for small object layers).

The suspicious objects identified are then input into the deep learning network model SAO-YOLO for further determination of whether they are abandoned objects. The proposed network model SAO-YOLO is illustrated in Figure 3, with CSPDarknet-53 as the backbone structure, and improvements made to address the characteristics of small objects and occluded objects.

Firstly, the network structure is adjusted to develop the SAO-FPN structure, which is advantageous for extracting features of small objects. Secondly, the proposed SODHead decoupled head is utilized, focusing on extracting local features and incorporating a self-attention mechanism to emphasize the features of occluded objects and small objects. This enhancement improves the model’s accuracy in detecting occluded objects and small objects.

Specifically, the input image is first normalized and resized to a uniform size of 640 × 640 × 3 (length × width × channels, where the length and width are in pixels). Next, the Backbone part, which has been streamlined, performs an initial feature extraction, resulting in a feature map of 40 × 40 × 512. Then, in the Neck part, a series of operations such as convolution, concatenation, upsampling, and pooling are conducted to produce three feature maps of 320 × 320 × 64, 160 × 160 × 128, and 80 × 80 × 256. Finally, the obtained feature maps are passed through the SODHead decoupled head for prediction and classification. P1, P2, and P3 represent the three different scales, with P1 and P2 retaining finer texture features, making them better suited for detecting medium and small objects.

#### 2.2.1. Feature Extraction Network SAO-FPN

**Figure 4:**
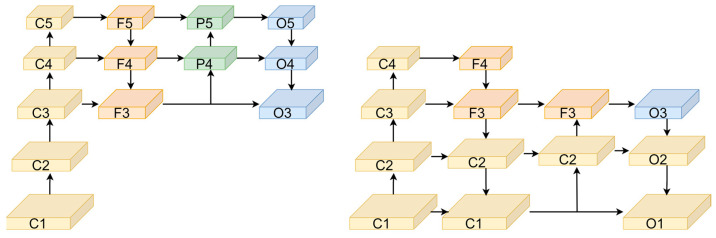
(**left**) The YOLOv5 feature extraction network structure diagram; (**right**) SAO-YOLO feature extraction network structure diagram. The proposed SAO-FPN feature extraction network reduces information loss by decreasing the overall network depth and adjusts the detection branches. By adding layers for small targets, it enhances the feature extraction capability for small objects.

In general object detection datasets, objects smaller than 32 × 32 pixels are typically classified as small objects. To enhance feature extraction for small objects, this paper increases the depth of the feature pyramid network, adjusts the scale of detection layer feature maps, and removes redundant modules, creating a feature extraction network centered around SAO-FPN. Specifically, during the downsampling process in the Backbone, to prevent loss of detail due to overly deep network structures, the Backbone has been simplified by removing the C5 module, resulting in a final feature map size of 40 × 40 × 512, which is then fed into the Feature Pyramid Module (SPPF) for further extraction. During the upsampling process in the Neck, to accommodate adjustments in the scale of detection layers, the depth of the feature pyramid network is increased, generating feature maps with 128 channels at 160 × 160 and 64 channels at 320 × 320, labeled F2 and F1 respectively. These are integrated with the main network’s C2 and C1 layers and matched in terms of channels and size, after which the combined 320 × 320 × 64 feature map F1, along with the outputs of P2 and P3, undergo classification, regression, and prediction in the Head section. Within the Neck’s Path Aggregation Network (PAN), to reduce unnecessary computation, the original output feature maps sized 40 × 40 × 512 (P4) and 20 × 20 × 1024 (P5) have been removed. The improved network structure, compared to the original structure, is illustrated in Figure 4.

#### 2.2.2. Implementation of SODHead

**Figure 5:**
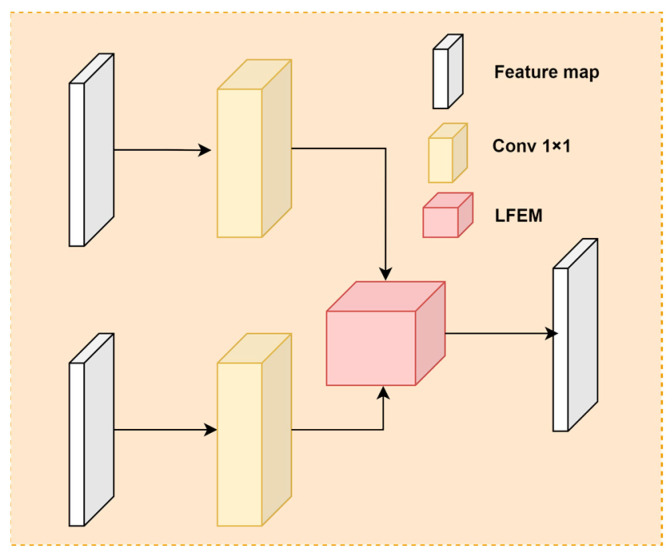
Basic structure diagram of the SODHead. SODHead mainly consists of 1 × 1 convolutions and the LFEM module. The high-level and low-level feature maps are preprocessed separately using 1 × 1 convolutions before being input into the LFEM module, which outputs the final feature map.

The decoupled head of YOLOv5 handles classification, regression, and prediction tasks in the same way across different scales of detection layers, but the feature maps output by these layers differ in detail expression and semantic richness. Lower-scale detection layers, such as those outputting feature maps of 320 × 320 × 64, tend to have a higher resolution, retaining extensive texture features and detail information through continuous upsampling and connections with the Backbone, which are crucial for detecting occluded and small objects. For medium to high-scale detection layers, located deeper in the network and subjected to multiple convolutions and pooling operations, the feature maps inputted to the detection head are usually smaller but contain richer semantic information, important for understanding complex images and larger objects. Therefore, to enhance the model’s ability to extract features from occluded and small objects while ensuring the existing multi-scale detection performance, this paper proposes a new type of decoupled head, SODHead, as shown in Figure 5. The basic structure includes convolution operations and the newly proposed LFEM (Local Feature Extraction Module). Initially, feature maps from two different detection layers are input in parallel, and 1 × 1 convolutions adjust each feature map’s channel size to match them, after which they are input into the LFEM module for local feature extraction and fusion, finally producing results for classification, regression, and prediction.

**Figure 6:**
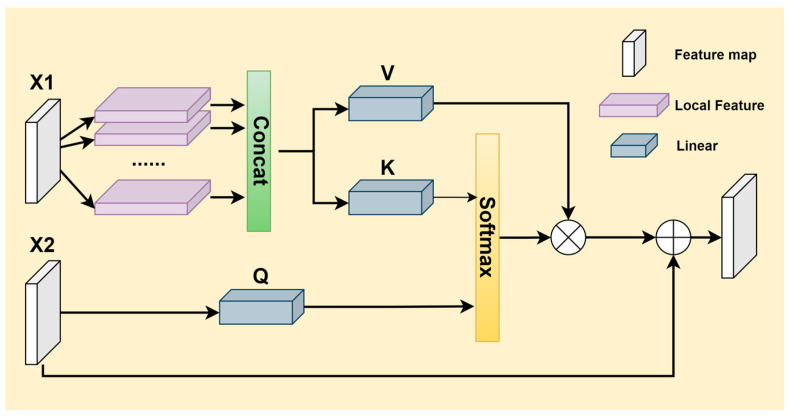
Basic structure diagram of the LFEM module. The LFEM module inputs the preprocessed X2 and X1 separately. After operations such as padding, cropping, and concatenation, X1 generates the corresponding V and K vectors, while X2 serves directly as the Q vector. The Q and K vectors are multiplied and normalized to compute the corresponding scores, which are then multiplied by the V vector. The result is fused with X2 to obtain the final output feature map.

High-level detection layers undergo extensive convolutions and pooling operations, providing rich semantic information that enhances the localization of detection targets. However, due to the loss of detailed features in deeper network layers, these levels are prone to misidentifications and missed detections of occluded and small objects. To address this issue, this paper designs an LFEM (Local Feature Extraction Module) that captures local features from lower detection layers for fusion. It simulates the self-attention mechanism transformations found in Transformer models to process input features, combining them with the output features of the high-level detection layers. This approach allows for precise extraction of feature information from occluded and small objects. The basic structure is illustrated in Figure 6.

Let the input from the lower detection layer be denoted as X1, and the input from the higher detection layer as X2. In the lower feature layer, X1 is first subjected to cropping, padding, and rearrangement operations to fully collect local feature information. Subsequently, two linear layers are used to construct vectors *K* and *V*, where *K* is used to calculate the similarity between features, and *V* is used to create a new output vector. In the higher feature layer, a linear layer is used to construct the *Q* vector, which captures essential information from the higher feature layer and provides a basis for calculating attention weights. The computed *Q* vector and *K* vector are then multiplied point-wise and normalized to compute attention scores, which are further normalized using operations like Softmax to produce the final attention weights. Finally, the normalized attention weights are used to perform a weighted summation of the *V* vector, which is then combined with *X2* to produce the final output feature map.

The main improvements are sequentially incorporated into the existing model, and the comparison of the improved model’s scale with the existing model’s scale is shown in Table 1.

## 3. Results

This section primarily evaluates the proposed abandoned object detection method, which integrates the adaptive dual-background model with SAO-YOLO, using the ABODA dataset [22], PETS2006 dataset, AVSS2007 dataset [21], and VisDrone2019 dataset [34]. It includes an introduction to the datasets and experimental environment, an assessment of the experimental results of the proposed model compared to other advanced models, and a series of analyses through ablation experiments, comparison experiments, and visualization experiments.

### 3.1. Datasets

**Figure 7:**
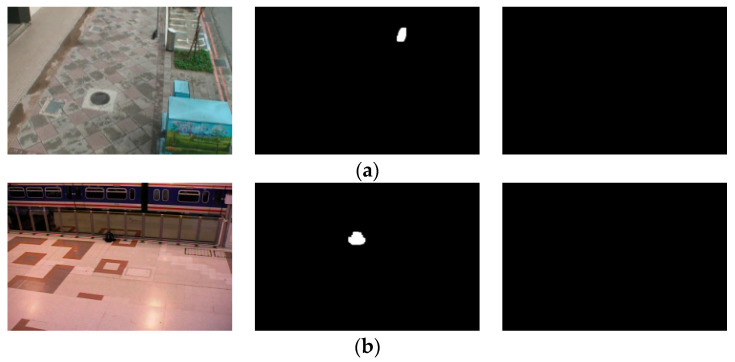
(**a**) The figure shows the original video frame, the long-term background model output, and the short-term background model output for the second scene in the ABODA dataset, and (**b**) shows the original video frame, the long-term background model output, and the short-term background model output for the first scene in the PETS2006 dataset.

This paper evaluates the adaptive dual-background model and the overall abandoned object detection method using the ABODA dataset and the PETS2006 dataset. The ABODA dataset consists of 11 scenarios, including a variety of settings such as indoor, outdoor, nighttime, and daytime. The PETS2006 dataset includes 7 different scenes and 2 temporarily abandoned scenes. The AVSS2007 dataset originates from the i-Lids dataset, primarily featuring scenes of abandoned luggage and parked vehicles. This study selects the abandoned luggage portion relevant to the research for experimental evaluation. The abandoned luggage consists of three video sequences, categorized by detection difficulty as AVSS AB Easy, AVSS AB Medium, and AVSS AB Hard. Similar to the PETS2006 dataset, each video sequence includes one abandonment event. Some of the output results are shown in Figure 7.

The VisDrone2019 dataset is collected and annotated by the AISKYEY team from the Machine Learning and Data Mining Lab at Tianjin University. As one of the most extensive and complex datasets available, it comprises 6471 training sets, 548 validation sets, and 1610 test sets, comprising a total of 2.6 million labels. The categories include ten types such as cars, pedestrians, buses, bicycles, tricycles, tricycles with canopies, trucks, vans, people, and motorcycles. The dataset contains a high proportion of small and occluded objects, particularly among pedestrians and people, presenting significant challenges. It also includes a vast amount of detail variation, making it highly suitable for training. Therefore, this paper selects this dataset to conduct experimental testing and evaluation of the SAO-YOLO model.

### 3.2. Environmental Configuration and Evaluation Metrics

The experiments were conducted on a machine equipped with an NVIDIA GTX 3060, using Windows 10 OS, with Python 3.9 as the programming language. The deep learning framework used was PyTorch 2.0.0 combined with CUDA 11.8, to implement the proposed detection model SAO-YOLO. The experiments were carried out for 300 epochs under consistent hyperparameter conditions, starting with a learning rate of 0.01.

The primary performance evaluation metrics used in this experiment include precision (*P*), recall (*R*), mean average precision (*mAP*), number of parameters (Params), and the amount of floating-point operations (GFLOPS). The formulas for these calculations are as follows:

(7) $$P=\frac{T_{P}}{T_{P}+F_{P}}$$

(8) $$R=\frac{T_{P}}{T_{P}+F_{N}}$$

(9) $$AP=\int_{0}^{1}P(R)dr$$

(10) $$mAP=\frac{\sum_{i=1}^{n}AP(i)}{n}$$

*T_P_* (True Positives) represents the number of predicted bounding boxes that meet the threshold requirement for the Intersection over Union (IoU) value with the true labels. *F_P_* (False Positives) refers to the number of detections where the IoU value does not meet the threshold requirement, i.e., the number of false detections. *F_N_* (False Negatives) represents the number of true labels that were not detected, i.e., the number of missed detections.

### 3.3. Experimental Results

#### 3.3.1. Adaptive Dual-Background Model Visualization Experiment

Changes in lighting and object complexity in abandoned object detection scenarios significantly impact the accuracy of detection, especially affecting small and occluded objects. To address this issue, this paper introduces lighting change factors and object complexity change factors in the background model to enhance the model’s adaptability to scene changes and improve the accuracy of the background model.

**Figure 8:**
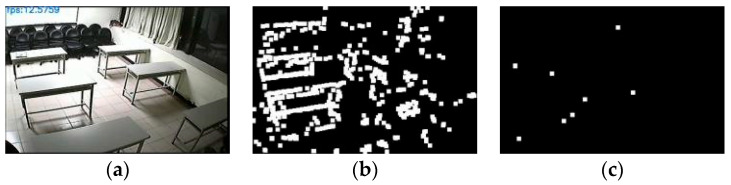
(**a**) The original frame during a lighting change in the 7th scene of the ABODA dataset, (**b**) The output result of the traditional mixture of Gaussians model for the same frame in this scene, (**c**) The output result of the adaptive mixture of Gaussians model with the lighting change factor introduced for the same frame in this scene.

To validate the effectiveness of the lighting change factor, experiments were conducted using the ABODA dataset, with some of the results shown in Figure 8. The adaptive mixture of Gaussians model on the right adjusts the learning rate using the lighting change factor. Compared to the traditional Gaussian model in the middle of the figure, it demonstrates better robustness to interference. This allows the model to adapt more quickly to lighting changes, reducing the impact of lighting variations on the model and improving detection accuracy.

**Figure 9:**
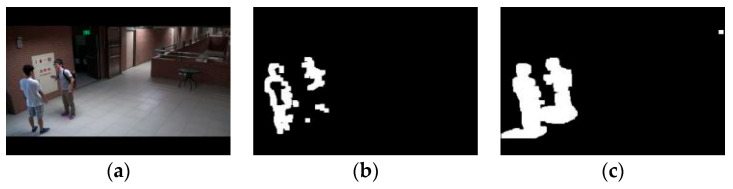
(**a**) The original frame from the first scene of the ABODA dataset, (**b**) The output result of the traditional mixture of Gaussians model for the same frame in this scene, (**c**) The output result of the adaptive mixture of Gaussians model for the same frame in this scene.

To validate the effectiveness of the target complexity change factor, experiments were conducted using the ABODA dataset, with some results shown in Figure 9. At the current moment, due to the high complexity and slow movement of the target object, the traditional mixture of Gaussians model in the middle image mistakenly classifies some pixels of the target as background. This results in the loss of some pixels during the target extraction process, leading to partially missing target objects. In contrast, the adaptive mixture of Gaussians model in the right image promptly adjusts the learning rate, thereby better extracting the target pixels.

#### 3.3.2. Evaluation of the Improved PFSM Model

To verify the effectiveness of the improved PFSM model for abandoned detection, this study employs the PETS2006 and AVSS2007 datasets for evaluation. PFSM-only is defined as the method that uses only the original PFSM model for abandoned detection after establishing the adaptive dual-background model, while Improved-PFSM-only is defined as the method that uses the improved PFSM model for abandoned object detection under the same conditions. The evaluation is conducted using precision and recall metrics.

From Table 2, it can be seen that in the PETS2006 dataset, the detection accuracy of the improved PFSM model is 63.6%, an improvement of 5.3% over the original model. In Table 3, the detection accuracy of the improved PFSM model is 50%, an improvement of 7% over the original model. In the datasets, the improved PFSM model has a certain improvement in complex scenes with occluded objects, but it is still prone to false alarms for stationary pedestrians due to its inability to judge the type of stationary objects. By using the complete residual object detection method proposed in this paper, the false alarm rate can be effectively reduced, with detection accuracy reaching 87.5% and 100%, respectively.

#### 3.3.3. SAO-YOLO Detection Scale Comparison Experiment

Without increasing the model size by adding detection branches, this experiment adjusts multi-scale detection on the basis of the original model, using a uniformly streamlined Backbone network and conducting quantitative analysis, with adjustments primarily focused on the Neck part. For ease of documentation, the detection branches in this experiment are divided into six levels according to the size of the output feature maps from the detection layers, named as O0, O1, O2, O3, O4, and O5, respectively. The detection layer represented by O1 outputs feature maps of size 640 × 640. The experimental data is from the VisDrone dataset, and the results are shown in Table 4.

From Table 4, it is evident that replacing O5 with O2 significantly improves the detection accuracy of the model for small object datasets, and a similar effect is achieved when further replacing O4 with O1. This improvement is due to the large-scale feature maps output by the lower detection layers containing more detail features, which more accurately capture the shape, size, and other information of small objects. When further adjustments are made to the detection scales, replacing them with higher resolution feature maps O0, the detail information contained in the feature maps is not much different from O1, and it increases the model’s burden. Therefore, this paper selects O1, O2, and O3 as the detection scales to implement multi-scale detection in the model.

#### 3.3.4. SAO-YOLO Ablation Study on Improvements

To better demonstrate the effectiveness of the modules and improvements proposed in this paper for detecting small and occluded objects, as well as their impact on parameters and floating-point computation, an ablation study was conducted, with results shown in Table 5. The experiment used YOLOv5 as a baseline, progressively adding the proposed modules and improvements. Initially, layer O5 in the model was replaced with O2, and the model was named M1. Subsequently, layer O4 was replaced with O1, naming the model M2. Then, the model’s Backbone and Neck were simplified, resulting in model M3. Finally, the original model’s head was replaced with the SODHead decoupled head, culminating in the final SAO-YOLO model.

As shown in Table 5, the proposed modules and improvements in this paper significantly enhance the detection accuracy of small and occluded objects. First, when adjusting the detection scale by replacing O5 with O2, the accuracy improves markedly, with mAP@0.5 increasing by 4.9 percentage points and mAP@0.5:0.95 by 3.0 percentage points. This indicates that the baseline model lacks detailed feature information, and the feature maps output by the lower detection layers can effectively compensate for the shortcomings in the baseline model regarding the size and shape information of small and occluded objects, thus improving the model’s detection accuracy. Next, replacing O4 with O1 further increases accuracy, with mAP@0.5 and mAP@0.5:0.95 improving by 2.6 and 1.3 percentage points, respectively, by better utilizing the feature texture information of small and occluded objects through further adjustments in detection scale. Then, simplifying the Backbone and Neck reduces the model’s parameters and floating-point computations by 5.6 and 6.0, respectively, while increasing mAP@0.5 and mAP@0.5:0.95 by 0.7 and 0.3 percentage points. This indicates that the original network lost some texture information of the detection objects during convolution, connection, and pooling operations. By appropriately reducing the network’s depth, it is possible to retain much of the original network model’s semantic information, reduce the loss of detailed feature information in the feature maps, and minimize unnecessary computations. Finally, adding the proposed SODHead decoupled head increases mAP@0.5 and mAP@0.5:0.95 by 0.8 and 0.5 percentage points, respectively. This shows that the SODHead decoupled head, by extracting local features, can further compensate for the information loss in high-level detection layers after multiple information fusions, preserving as much critical feature information of occluded and small objects as possible, thereby improving detection accuracy.

#### 3.3.5. Comprehensive Comparison Experiment of the SAO-YOLO Model

Finally, to comprehensively demonstrate the detection effectiveness of the proposed model, several advanced mainstream models were selected and compared with SAO-YOLO and the baseline model. The experiment used the VisDrone dataset, with 300 training epochs, and the final test results were validated on the VisDrone2019 dataset, as shown in Table 6.

This section mainly analyzes mAP@0.5 and the number of parameters as evaluation metrics. From the table, it can be seen that the improved model proposed in this paper has a significant advantage in detection accuracy despite having a far smaller parameter scale compared to other mainstream models. The proposed model significantly outperforms classic models like SSD and Faster-RCNN in detection accuracy, with mAP@0.5 being 19.4 and 9.7 percentage points higher, respectively. Compared to other models in the YOLO series, including YOLOv3, YOLOv4, YOLOv5s, YOLOv6, YOLOv7, and YOLOv8s, the proposed model also has a notable advantage in parameter scale, with overall mAP@0.5 being 7.5%, 7.1%, 9.0%, 8.7%, 2.6%, and 2.1% higher, respectively. Although the TPH-YOLOv5 model shows superior performance in detecting small and occluded objects within the YOLO series, the proposed model in this paper still surpasses it in mAP@0.5 and mAP@0.5:0.95 by 1.1% and 3.0%, respectively, while also having a lower parameter scale.

#### 3.3.6. SAO-YOLO Visualization Experiment

**Figure 10:**
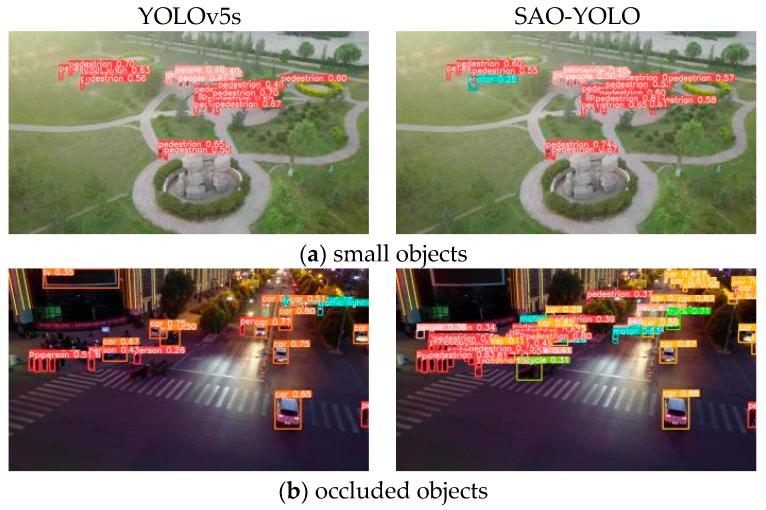
Visual comparison of experimental results. (**a**,**b**) are both sourced from the VisDrone dataset, where Figure (**a**) illustrates the detection performance for small objects, and Figure (**b**) shows the detection performance for occluded objects.

From the aforementioned experimental results, it is evident that the enhanced SAO-YOLO model not only excels other mainstream models in detecting small and occluded objects but also possesses a more lightweight overall model size. Figure 10 presents a comparison of the effects between the SAO-YOLO model and the baseline model.

Complex scenarios of abandoned object detection frequently entail issues like small objects and objects occluded by crowds. In the comparison of small object detection presented in Figure 10a, the baseline model misses the motorcycle beside the pedestrian, while the improved SAO-YOLO model shows no missed detections. Dense occlusion is a common occurrence in abandoned object detection scenes, as depicted in Figure 10b. The baseline model fails to identify partially occluded pedestrians, but the SAO-YOLO model effectively overcomes this limitation, leading to enhanced detection accuracy. In contrast, the SAO-YOLO model significantly reduces missed detections in such scenarios.

#### 3.3.7. Abandoned Object Detection Experiment

To validate the efficacy of the abandoned object detection approach proposed in this paper, experiments were carried out by applying this method to 11 scenarios within the ABODA dataset. The experimental data encompasses scenario number, the quantity of abandoned objects, (the number of accurately identified positive samples), (the number of wrongly identified negative samples), and scenario type. The experimental outcomes are presented in Table 7. The results demonstrate that the overall recall rate of the method amounts to 100%, and the overall detection accuracy is 85.7%.

This paper makes a comparison among several advanced methods, with the results on the ABODA dataset presented in Table 8. The proposed abandoned object detection method attained a recall rate (R) of 100% and a precision (P) of 85.7% on the ABODA dataset. In terms of both recall and precision, the proposed method either meets or exceeds other advanced abandoned object detection methods, demonstrating superior detection performance.

Due to the diverse nature of real-world scenarios, factors such as the form, location, and environmental conditions of observations can significantly influence the results. Therefore, relying solely on the ABODA dataset to evaluate the accuracy of the proposed abandoned object detection methods presents certain limitations. To enhance the scientific validity of our statistical data, this paper further validates the method using both the PETS2006 dataset and the AVSS2007 dataset. We also compare these results with those obtained from ABODA by calculating their averages, thus increasing the reliability of our experimental findings. The outcomes are presented in Table 9 and Table 10.

In this study, the ABODA, PETS2006, and AVSS2007 datasets were utilized to perform abandoned object detection experiments, with the results averaged as presented in Table 10. The recall rate and precision of this method are recorded at 100% and 91.1%, respectively, indicating a precision improvement of 2.2% over that reported by Lin et al. This finding further substantiates the efficacy of this approach.

#### 3.3.8. Experimental Evaluation of the Detection Error of Abandoned Object

To estimate the error of the parameters, this paper trains the SAO-YOLO model using a random grouping method. Specifically, the experiment uses the VisDrone2019 dataset for the experiment, and the training set, validation set, and test set in the dataset are randomly divided into two parts to obtain 3235 training set samples, 274 validation set samples, and 805 test set samples, respectively, and trained separately to obtain their respective training results. To ensure the quality of the random grouping training, this paper will conduct three random grouping experiments, training the model a total of six times, and the experimental results are shown in Table 11.

Based on the data presented in Table 11, it is evident that the minimum mAP@0.5 value across six model training scenarios is 37.8%, while the maximum reaches 38.9%, resulting in an interquartile range of 1.1%. In contrast, the minimum mAP@0.5:0.95 value stands at 20.5%, with a maximum of 21.2% and an interquartile range of just 0.7%. The mean Average Precision (mAP) serves as a crucial metric for assessing the performance of target detection models, particularly under varying Intersection over Union (IoU) threshold values. Specifically, mAP@0.5 denotes the average precision achieved by the model when the IoU threshold is set to 0.5, whereas mAP@0.5:0.95 reflects average precision performance across multiple IoU thresholds. Notably, the variation in mAP@0.5 is limited to only 1.1%, while that for mAP@0.5:0.95 is even more constrained at just 0.7%. Analysis of multiple experimental results reveals consistently small errors, indicating that the SAO-YOLO model demonstrates highly stable detection performance under both evaluation criteria.

To better illustrate the errors in abandoned object detection performance, each trained model group is integrated into the overall abandoned object detection method, and the comprehensive ABODA dataset is utilized for detection. The resulting experimental outcomes are shown in Table 12.

Based on the data analysis of Table 12, the recall rate of the 6 random group training datasets reached 100%, while the precision rate fluctuated between a maximum of 75% and a minimum of 71%, with a minor error. This outcome further validates the stability of the SAO-YOLO model.

## 4. Conclusions

To address the issues of false positives and missed detections in existing methods for detecting small and occluded objects, we propose a novel abandoned object detection approach that integrates an adaptive double-background model with SAO-YOLO. This model enhances adaptability to scene variations and noise, while the improved PFSM model effectively extracts suspicious objects (i.e., static foreground elements). Experimental results on the ABODA, PETS2006, AVSS2007, and VisDrone2019 datasets demonstrate that the combination of the dual-background model and SAO-YOLO significantly improves detection accuracy for small- to medium-sized objects as well as occluded objects in abandoned target scenarios, achieving an average detection accuracy of 91.1%, which is markedly superior to other advanced detection technologies.

At present, accurately detecting small and occluded objects in abandoned object scenes continues to be a considerable challenge. There is still a scarcity of datasets specifically tailored for abandoned object scenes, including those customized for the categories essential for effective detection and those concentrating on abandoned object identification. As a result, the experimental results presented in this paper might not comprehensively reflect the accuracy of complex real-world scenarios related to abandoned object detection and have certain limitations. Additionally, diverse factors such as video acquisition approaches, locations, and environmental conditions can impact actual detection outcomes in real-world scenarios, presenting further challenges to the application of abandoned object detection techniques.

## Figures and Tables

**Table 1:** Comparison of the improved network size with the original network model.

Structure	Input Size	Param/M	Gflops
YOLOv5s	640 × 640 × 3	7.0	15.8
YOLOv5s + SAO-FPN	640 × 640 × 3	1.6	15.6
YOLOv5s + SAO-FPN + SODHead	640 × 640 × 3	1.7	18.2

**Table 2:** Performance comparison of the PFSM model on the PETS2006 dataset.

Method	R/%	P/%
PFSM-only	100	58.3
Improved-PFSM-only	100	63.6
Improved-PFSM with SAO-YOLO (Ours)	100	87.5

**Table 3:** Performance comparison of the PFSM model on the AVSS2007 dataset.

Method	R/%	P/%
PFSM-only	100	43
Improved-PFSM-only	100	50
Improved-PFSM with SAO-YOLO (Ours)	100	100

**Table 4:** Quantitative analysis of multiscale detection.

Detection Branch	mAP@0.5/%	mAP@0.5:0.95/%	Param/M
O3, O4, O5	34.5	19.2	15.8
O2, O3, O4	39.4	22.2	18.7
O1, O2, O3	42.0	23.5	21.6
O0, O1, O2	42.1	23.6	22.8

**Table 5:** Ablation experiments on the VisDrone dataset.

Structure	mAP@0.5/%	mAP@0.5:0.95/%	Param/M	GFLOPS
Baseline	34.5	19.2	7.0	15.8
M1	39.4	22.2	7.1	18.7
M2	42.0	23.5	7.2	21.6
M3	42.7	23.8	1.6	15.6
SAO-YOLO	43.5	24.3	1.7	18.2

**Table 6:** Comprehensive comparison experiment of the SAO-YOLO model.

Structure	P/%	R/%	mAP@0.5/%	mAP@0.5:0.95/%	Param/M
SSD [13]	21.1	35.6	24.1	18.8	24.5
Faster-RCNN [15]	43.3	35.6	33.8	21.4	41.29
YOLOv3 [9]	50.3	37.4	36.0	19.4	63.07
YOLOv4 [10]	47.9	39.8	36.4	20.1	61.4
YOLOv5s	46.8	34.5	34.5	19.2	7.0
YOLOv6 [11]	44.6	38.5	34.8	18.5	9.67
YOLOv7 [12]	53.5	42.5	40.9	22.3	37.2
YOLOv8s	53.3	40.0	41.4	25.1	11.1
TPH-YOLOv5 [35]	51.1	39.2	42.4	21.3	22.5
Ours	54.2	41.4	43.5	24.3	1.7

**Table 7:** ABODA Dataset abandoned object detection results.

Scene Number	Abandoned Object	TP	FP	R/%	P/%
Video1	1	1	0	100	85.7
Video2	1	1	0		
Video3	1	1	0		
Video4	1	1	0		
Video5	1	1	0		
Video6	2	2	0		
Video7	1	1	0		
Video8	1	1	0		
Video9	1	1	0		
Video10	1	1	0		
Video11	1	1	2		

**Table 8:** Comparison of detection methods for abandoned objects on the ABODA dataset.

Methods	TP	FP	R/%	P/%
Ours	12	2	100	85.7
Saluky et al. [21]	9	3	75	75
Ilya et al. [23]	9	4	75	69.2
Lin et al. [22]	12	6	100	66.7

**Table 9:** Comparison of abandoned object detection methods on PETS2006 dataset and AVSS2007 dataset.

	PETS2006		AVSS2007	
Methods	R/%	P/%	R/%	P/%
Ours	100	87.5	100	100
Saluky et al. [21]	70	77	72	72
Ilya et al. [23]	/	/	/	/
Lin et al. [22]	100	100	100	100

**Table 10:** Comprehensive comparison results of abandoned object detection methods.

Methods	R/%	P/%
Ours	100	91.1
Saluky et al. [21]	72.3	74.7
Ilya et al. [23]	/	/
Lin et al. [22]	100	88.9

**Table 11:** Random grouping experiments of the SAO-YOLO model on the VisDrone2019 dataset.

Experiment Group	Split Method	mAP@0.5/%	mAP@0.5:0.95/%
Group 1	Random Split 1	38.4	21.2
	Random Split 2	38.7	21.1
Group 2	Random Split 1	38.1	20.9
	Random Split 2	37.8	20.5
Group 3	Random Split 1	38.9	21.2
	Random Split 2	38.1	20.7

**Table 12:** Abandoned object detection experiments using the SAO-YOLO model trained with randomly grouped datasets.

Experiment Group	Split Method	R/%	P/%
Group 1	Random Split 1	100	75
	Random Split 2	100	75
Group 2	Random Split 1	100	71
	Random Split 2	100	71
Group 3	Random Split 1	100	75
	Random Split 2	100	71

## Data Availability

The datasets used in this paper are all publicly available datasets, which are composed of three datasets in total. Here are the links to the two publicly availabledatasets: https://github.com/kevinlin311tw/ABODA (accessed on 29 August 2024), and http://aiskyeye.com/download/ (accessed on 29 August 2024). Another dataset, for unknown reasons, is not currently available. After downloading them, the authors made datasets suitable for usethrough their own annotations. The data presented in this study are available on request from thecorresponding author.
